# Effects of Different Types of *Lactobacillus helveticus* Exopolysaccharides on Immune Function in Immunodeficient Mice

**DOI:** 10.3390/foods15020261

**Published:** 2026-01-11

**Authors:** Shunyu Wang, Hongchao Wang, Fuhao Li, Yurong Zhao, Zhangming Pei, Wenwei Lu, Jianxin Zhao, Shourong Lu

**Affiliations:** 1 State Key Laboratory of Food Science and Resources, Jiangnan University, Wuxi 214122, China; 7200112089@stu.jiangnan.edu.cn (S.W.); hcwang@jiangnan.edu.cn (H.W.); leefh123@163.com (F.L.); 6230111151@stu.jiangnan.edu.cn (Y.Z.); peizhangming@jiangnan.edu.cn (Z.P.); luwenwei@jiangnan.edu.cn (W.L.); 2 School of Food Science and Technology, Jiangnan University, Wuxi 214122, China; 3 Zhejiang Liziyuan Food Co., Ltd., Jinhua 321031, China; 4 The Affiliated Wuxi People’s Hospital of Nanjing Medical University, Wuxi People’s Hospital, Wuxi Medical Center, Nanjing Medical University, Wuxi 214122, China

**Keywords:** lactobacillus exopolysaccharides, mannose, fucose, gut microbiota, immunomodulation

## Abstract

Immunodeficiency presents a significant clinical challenge in contexts such as tumour radiotherapy, chemotherapy, and organ transplantation. Current therapeutic interventions are constrained by single-target approaches and substantial adverse effects. As natural bioactive compounds, the immunomodulatory activities of *Lactobacillus* exopolysaccharides (EPS) are intimately linked to their monosaccharide composition. Mannose and fucose, two rare functional monosaccharides, fulfil critical roles in physiological processes including immune recognition and inflammatory regulation. However, the functional optimisation of EPS through mannose and fucose enrichment remains incompletely characterised. This study established a cyclophosphamide (CTX)-induced immunodeficient mouse model to investigate the immunomodulatory effects of mannose-enriched and fucose-enriched EPS derived from *Lactobacillus helveticus*. Intervention efficacy was evaluated through a comprehensive assessment of immune organ indices, cytokine profiles, histopathological alterations, and gut microbiota composition. Both mannose-enriched and fucose-enriched EPS significantly elevated splenic indices and ameliorated white pulp atrophy. Furthermore, these EPS variants restored cytokine homeostasis in serum and small intestinal tissues, attenuated hepatic steatosis, and restructured the gut microbiota by enhancing microbial diversity, increasing Firmicutes abundance, and elevating the relative proportions of Bacteroides, Faecalibacterium, and Bifidobacterium. Collectively, mannose-enriched and fucose-enriched EPS from *Lactobacillus helveticus* alleviated CTX-induced immunodeficiency through multiple mechanisms, including restoration of immune organ integrity, modulation of cytokine networks, and re-establishment of gut microbiota homeostasis. This study provides a theoretical foundation for developing immunomodulatory functional foods and offers novel insights into the microbiota-immunity axis in immune regulation.

## 1. Introduction

The immune system constitutes the body’s principal defence mechanism against exogenous pathogens whilst maintaining internal homeostasis; its dysfunction or compromise can precipitate the onset and progression of numerous pathological conditions. In recent years, the escalating incidence of cancer chemotherapy and radiotherapy, immunosuppressive regimens for organ transplantation, and autoimmune disorders has rendered immunodeficiency-related complications a formidable clinical challenge [1,2]. Cyclophosphamide (CTX), a widely employed alkylating immunosuppressant, exerts its therapeutic effects by inhibiting lymphocyte proliferation and differentiation. However, its clinical application is accompanied by substantial adverse effects, including immune organ damage, cytokine network disruption, and gut microbiota dysbiosis, thereby significantly compromising patients’ quality of life [3]. Consequently, the identification of safe and efficacious natural immunomodulators to ameliorate immunodeficient states has emerged as a research priority.

*Lactobacillus*, a pivotal probiotic genus within the human gastrointestinal tract, has attracted considerable attention for its metabolic product—exopolysaccharides (EPS)—owing to their remarkable biological activities and favourable safety profiles. Lactic acid bacteria EPS comprise a class of high-molecular-weight carbohydrates secreted extracellularly during bacterial growth and metabolism. Their chemical architectures exhibit substantial complexity and diversity, with monosaccharide composition, glycosidic linkage patterns, and molecular weight distribution collectively influencing their biological activities [4,5]. Notably, mannose and fucose—two rare functional monosaccharides—fulfil pivotal roles in physiological processes encompassing immune recognition and inflammatory regulation [6]. We identified 28 strains of *Lactobacillus helveticus* with immunomodulatory functions from the microbial database featuring in vitro probiotic properties (https://probio-ichnos.streamlit.app/ (accessed on 23 December 2025)) [7]. For instance, *Lactobacillus helveticus* R0052 inhibits the adhesion of Escherichia coli O157:H7 to epithelial cells; *Lactobacillus helveticus* HY7801 reduces the levels of pro-inflammatory cytokines; and *Lactobacillus helveticus* MIMLh5 exerts antagonistic effects against Streptococcus pyogenes. However, few studies have reported investigating the impact of different types of exopolysaccharides (EPS) on immunomodulation by altering the culture conditions to induce *Lactobacillus helveticus* to produce diverse EPS subtypes. Therefore, we attempted to optimise the culture conditions and explore the immunomodulatory effects of exopolysaccharides produced by *Lactobacillus helveticus* DYNDL_20-5 and *Lactobacillus helveticus* 6M-3.

Preliminary investigations have demonstrated that supplementation of culture media with specific monosaccharides can selectively modulate the monosaccharide composition of *Lactobacillus* EPS. Specifically, mannose supplementation substantially increased mannose content in EPS from *Lactobacillus helveticus* 6M-3, whilst fucose addition enhanced fucose incorporation in EPS from *Lactobacillus helveticus* DYNDL_20-5. These findings provide novel perspectives for optimising the immunomodulatory functions of EPS.

During immunodeficient states, the dynamic equilibrium between the host immune system and gut microbiota becomes disrupted, establishing a detrimental cycle of immune suppression → dysbiosis → further immune deterioration. As the largest microbial ecosystem in the human body, the gut microbiota participates in essential physiological processes, including immune cell maturation, cytokine secretion, and mucosal barrier maintenance, through its metabolic products such as short-chain fatty acids and lipopolysaccharides [8,9]. Evidence confirms that immunodeficient mice exhibit markedly reduced gut microbiota diversity and altered dominant microbial community structures. Among these alterations, perturbations in the Firmicutes-to-Bacteroidetes ratio are considered a cardinal indicator of aberrant immune function [10,11]. Nevertheless, the precise mechanisms whereby *Lactobacillus* EPS ameliorates immunodeficiency through gut microbiota modulation remain incompletely elucidated. Particularly, the regulatory effects of monosaccharide-optimised EPS on the microbiota-immunity axis warrant systematic investigation.

Building upon this research foundation, the present study innovatively employs a culture medium optimisation strategy to generate *Lactobacillus* EPS enriched with either mannose or fucose. Utilising CTX-induced immunodeficient mice as an experimental model, this investigation systematically evaluates the immunoregulatory effects of monosaccharide-modified EPS. The experimental framework encompasses a multidimensional assessment system, including immune organ index measurements, cytokine profile analyses, histopathological examinations of splenic and hepatic tissues, and comprehensive characterisation of gut microbiota diversity and compositional architecture. By comparing the immunomodulatory activities of EPS derived from conventional versus monosaccharide-optimised culture conditions, this study elucidates the intrinsic relationships amongst monosaccharide composition, gut microbiota restructuring, and immune function restoration. These findings provide experimental evidence and theoretical support for developing novel *Lactobacillus* EPS-based immunomodulators, whilst offering new research directions for nutritional intervention strategies targeting immunodeficiency-related disorders.

## 2. Materials and Methods

### 2.1. Materials and Reagents

Peptone, yeast extract, anhydrous sodium acetate, diammonium hydrogen citrate, K_2_HPO_4_, magnesium sulphate heptahydrate, L-cysteine hydrochloride, beef extract, anhydrous D-glucose, Tween 80, manganese sulphate monohydrate, sodium chloride, and manganese sulphate tetrahydrate were obtained from China National Pharmaceutical Group Chemical Reagent Co., Ltd. (Shanghai, China). Paraformaldehyde fixative (4%) was purchased from Shanghai Beyotime Biotechnology Co., Ltd. (Shanghai, China). Enzyme-linked immunosorbent assay (ELISA) kits for detecting murine interleukin (IL)-1β, IL-6, IL-10, tumour necrosis factor (TNF)-α, and interferon (IFN)-γ were acquired from Shanghai Enzyme-linked Biotechnology Co., Ltd. (Shanghai, China).

### 2.2. Instruments and Equipment

Magnetic stirrer (IKA, Staufen, Germany); vacuum freeze dryer (LABCONCO, Kansas City, MO, USA); microplate reader (Thermo Fisher Scientific, Waltham, MA, USA); FE20 pH metre (Mettler Toledo Instruments, Shanghai, China); EL3002 electronic balance (Mettler Toledo Instruments, Shanghai, China); ultra-low temperature freezer ULT1386-3-V31 (Thermo Fisher Scientific, USA); MS3 basic vortex mixer (IKA, Germany); fume hood (Yangzhou Tianhui Steel and Wood Products Co., Ltd., Yangzhou, China); Model 5415 and Model 5804R refrigerated high-speed centrifuges (Eppendorf GmbH, Hamburg, Germany); Model MLS-3750 high-temperature high-pressure autoclave (Sanyo Electric Co., Ltd., Osaka, Japan); Model ZHJH-C1115B laminar flow cabinet (Shanghai Zhicheng Analytical Instrument Manufacturing Co., Ltd., Shanghai, China); Milli-Q water purification system (Millipore, Shanghai, China).

### 2.3. Experimental Methods

#### 2.3.1. Extraction of Exopolysaccharides

Inoculate the bacterial strain into MRS liquid medium at an inoculum size of 4% (*v*/*v*), culture at 37 °C for 24 h, and perform two consecutive subcultures until viability is restored. Transfer 4% (*v*/*v*) of the activated bacterial suspension into 100 mL of MRS liquid medium and conduct anaerobic culture at 37 °C for 48 h. Subject the fermentation broth to a boiling water bath for 20 min; after cooling to room temperature, centrifuge (4 °C, 8000 rpm, 15 min) to remove bacterial cells and collect the supernatant. Add trichloroacetic acid (80% *w*/*v*) to the supernatant to achieve a final concentration of 4%, and incubate at 4 °C for 24 h. Centrifuge the trichloroacetic acid-treated solution (4 °C, 10,000 rpm, 20 min) and collect the supernatant. Add absolute ethanol at a volume three times that of the supernatant and incubate at 4 °C for 48 h. Following incubation, centrifuge (4 °C, 10,000 rpm, 20 min), collect the precipitate, and redissolve in ultrapure water. Dissolve the crude polysaccharide precipitate in deionised water at 60 °C; after complete dissolution, centrifuge (4 °C, 10,000 rpm, 10 min) to remove insoluble material. Load the supernatant into an ultrafiltration tube for purification. Prepare the dialysate into lyophilised powder using a freeze-dryer and store at −20 °C.

#### 2.3.2. Monosaccharide Composition and Proportional Analysis of Extracellular Polysaccharides

Dissolve 5 mg of Lactobacillus extracellular polysaccharide in 1 mL of trifluoroacetic acid (4 M) and hydrolyse at 121 °C for 3.5 h. After cooling, evaporate the remaining trifluoroacetic acid under nitrogen. Resuspend the solid residue in ultrapure water and filter through a 0.22 μm membrane. Determine the monosaccharide composition of extracellular polysaccharides using high-pressure ion chromatography (Dionex ICS-5000, Thermo Fisher Scientific, CA, USA) equipped with a Dionex CarboPac PA20 column (3 × 150 mm). The experimental conditions were as follows: column temperature of 30 °C; mobile phases consisting of ultrapure water, 1 M CH_3_COONa, and 0.25 M NaOH; flow rate of 0.5 mL/min.

#### 2.3.3. Animal Experiment Design

All experimental protocols were conducted in accordance with the European Community Guidelines (2010/63/EU) and approved by the Ethics Committee of Jiangnan University (JN. No.20231030c1040131[514]). The mice used were 6-week-old female C57BL/6J. Mice were housed in the Animal Research Centre at Jiangnan University under barrier conditions (temperature 20–26 °C, relative humidity 40–70%, 12 h light–dark cycle). Drinking water and cage equipment were changed weekly at the same time. Feed was replaced every 2 to 3 days. All mice had free access to food and water and were fed standard mouse chow. The modelling method was appropriately modified based on previous studies. After a one-week adaptation period, 8 mice were randomly assigned to the negative control group (NC group) and received no treatment, whilst the remaining 48 mice received intraperitoneal injections of cyclophosphamide (CTX) for 3 days. On Day 4, the 48 mice that received intraperitoneal injections of CTX were randomly divided into 6 groups of 8 mice each, designated as follows: positive control group (PC group), model group (MC group), extracellular polysaccharide *L. helveticus* DYNDL_20-5 (cultured in fucose-containing medium) group (L.H 20-5(T) group), extracellular polysaccharide *L. helveticus* 6M-3 (maltose-containing medium) group (L.H 6M-3(T) group), extracellular polysaccharide *L. helveticus* DYNDL_20-5 group (L.H 20-5 group), and extracellular polysaccharide *L. helveticus* 6M-3 group (L.H 6M-3 group). Eight mice in the PC group received intraperitoneal injections of thymosin for 7 days, whilst the remaining mice in each group were gavaged with their respective extracellular polysaccharides for 7 days. The NC and MC groups were gavaged with physiological saline for 7 days. Tissue samples were collected from experimental mice on Day 11.

#### 2.3.4. Measurement of Mouse Body Weight and Spleen Weight

After the experiment, all mice were weighed and euthanised by cervical dislocation. The spleen was removed, its surface dried with filter paper, and the spleen weight recorded. The spleen-to-body weight ratio was calculated as follows:

Spleen/Body Weight = Spleen Weight (mg)/Mouse Body Weight (g)

#### 2.3.5. Measurement of Mouse Colon Length

After the experiment, mice were euthanised by cervical dislocation. The mouse colon was excised, retaining the caecum portion. The caecal end was positioned at the 0 cm mark, and the colon length was measured.

#### 2.3.6. Haematoxylin–Eosin Staining

Tissues were fixed in 4% paraformaldehyde solution at 4 °C for 36 h, then dehydrated, cleared, embedded, and sectioned into 5 μm slices. Slides were stained with haematoxylin and eosin, followed by dehydration and clearing. Slides were observed and photographed using a confocal laser scanning microscope (Nikon, Tokyo, Japan).

#### 2.3.7. Cytokine Assay

Remove the required strips from the aluminium foil bag that has been equilibrated at room temperature for 60 min. Set up standard wells and sample wells: add 50 μL of standards at different concentrations to each standard well, add 50 μL of the sample to be tested to each sample well, and leave the blank well empty. Except for the blank well, add 100 μL of horseradish peroxidase (HRP)-labelled detection antibody to each standard well and sample well. Seal the reaction wells with a plate sealer and incubate in a 37 °C water bath or incubator for 60 min. Discard the liquid, blot the plate dry on absorbent paper, fill each well with 350 μL of washing solution, allow to stand for 1 min, discard the washing solution, and blot the plate dry on absorbent paper. Repeat the washing process five times. Add 50 μL of Substrate A and 50 μL of Substrate B to each well and incubate at 37 °C in the dark for 15 min. Add 50 μL of stop solution to each well and measure the optical density (OD) value of each well at a wavelength of 450 nm using a microplate reader.

#### 2.3.8. Mouse Small Intestinal Cytokine Assay

First, small intestinal tissue stored at −80 °C was retrieved and rinsed with pre-cooled PBS (0.01 M, pH 7.4) to remove residual blood and adipose tissue. Approximately 0.12 g of tissue (fresh weight) was weighed and cut into small pieces. The tissue grinder tray was pre-cooled in advance, and the cut tissue was transferred into an EP tube along with PBS (0.01 M, pH 7.4) at a tissue-to-buffer ratio of 1:9 (*w*/*v*), followed by the addition of grinding beads. The mixture was thoroughly homogenised in the tissue grinder, and the homogenate was subsequently centrifuged at 5000× *g* for 10 min. The supernatant was collected, aliquoted, and stored at −20 °C for subsequent analysis.

Microplate strips were removed from aluminium foil bags that had been equilibrated at room temperature for 60 min. Standard wells and sample wells were prepared as follows: 50 μL of standards at varying concentrations were added to each standard well, 50 μL of test sample was added to each sample well, and blank wells received no additions. Except for the blank wells, 100 μL of horseradish peroxidase (HRP)-conjugated detection antibody was added to all standard and sample wells. The reaction plate was sealed with a plate sealer and incubated at 37 °C for 60 min in a water bath or incubator. Following incubation, the liquid was discarded, and the plate was blotted dry on absorbent paper. Each well was filled with 350 μL of washing solution, allowed to stand for 1 min, and then emptied and blotted dry. This washing procedure was repeated five times. Subsequently, 50 μL each of Substrate A and Substrate B was added to each well, and the plate was incubated at 37 °C in the dark for 15 min. Finally, 50 μL of stop solution was added to each well, and the optical density (OD) value was measured at 450 nm using a microplate reader.

#### 2.3.9. Gut Microbiota 16S rDNA Analysis

Total bacterial DNA was extracted from faecal samples according to the manufacturer’s instructions using the Fast DNA Spin Kit for Feces (MP Biomedicals, Santa Ana, CA, USA). The V3–V4 region of the bacterial 16S rRNA gene was amplified by PCR using barcoded primers 338F (5′-ACTCCTACGGGAGGCAGCAG-3′) and 806R (5′-GGACTACHVGGGTWTCTAAT-3′). The PCR reaction mixture consisted of 25 μL of 2× Taq Plus MasterMix (Dye), 1 μL of each primer (338F and 806R), 1 μL of DNA template, and 22 μL of double-distilled water. The PCR cycling conditions were as follows: initial denaturation at 95 °C for 5 min; 30 cycles of denaturation at 95 °C for 30 s, annealing at 52 °C for 30 s, and extension at 72 °C for 30 s; followed by a final extension at 72 °C for 10 min.

PCR products were verified by electrophoresis on a 1.5% (*w*/*v*) agarose gel, and the target bands were excised and purified using a DNA Gel/PCR Purification Miniprep Kit. Purified amplicons were quantified, pooled in equimolar concentrations, and used for library construction. Sequencing was performed on an Illumina platform using the PE300 strategy (paired-end, 2 × 300 bp), generating an average of approximately 50,000 raw reads per sample.

Raw sequencing reads were demultiplexed according to sample-specific barcodes and trimmed to remove barcodes and primer sequences using Cutadapt (version 4.7) [12]. Subsequent bioinformatic analyses were conducted using QIIME2 (version 2022.2) [13]. The DADA2 plugin was applied for quality control of paired-end reads, including filtering of low-quality sequences, trimming, denoising, merging of paired-end reads, and removal of chimeric sequences using the consensus method, resulting in high-resolution amplicon sequence variants (ASVs). Taxonomic assignment of ASVs was performed by comparison against the SILVA ribosomal RNA gene database (version 132) [14].

Differential abundance analysis between groups was conducted using linear discriminant analysis effect size (LEfSe) to identify taxa that significantly contributed to group differences and to estimate their effect sizes. Statistical significance was first assessed using non-parametric tests with a threshold of *p* < 0.05, followed by linear discriminant analysis (LDA) to evaluate the magnitude of differences. Taxa with *p* < 0.05 and an LDA score > 2.0 were considered significantly different. LEfSe analysis was performed using the algorithm implemented on the Bioincloud platform (https://www.bioincloud.tech/ (accessed on 1 November 2025)) [15].

The sequencing data generated in this study have been deposited in the NCBI BioProject database under accession number PRJNA1381239.

#### 2.3.10. Data Analysis

Data are presented as mean ± SEM. Statistical analysis and graphical representation were performed using GraphPad Prism 9.0. One-way analysis of variance (ANOVA) was employed to assess differences between groups. For comparisons between experimental groups and the MC group, statistical significance was defined as * *p* < 0.05, ** *p* < 0.01, and *** *p* < 0.001.

## 3. Results

### 3.1. Effects of Mannose or Fucose Supplementation on the Extracellular Polysaccharide Composition of Lactobacillus helveticus

Preliminary investigations demonstrated that supplementation of culture media with specific monosaccharides can selectively modulate the monosaccharide composition of *Lactobacillus* EPS. Specifically, mannose supplementation significantly increased the mannose content in EPS produced by *Lactobacillus helveticus* 6M-3, whilst fucose supplementation enhanced fucose incorporation in EPS from *Lactobacillus helveticus* DYNDL_20-5. Following cultivation of *L. helveticus* strains in media supplemented with mannose or fucose, the composition and content of extracellular polysaccharides were analysed, with results presented in Table 1 and Table 2.

### 3.2. Effects of CTX and Extracellular Polysaccharide Intervention on Murine Immunity

**Figure 1:**
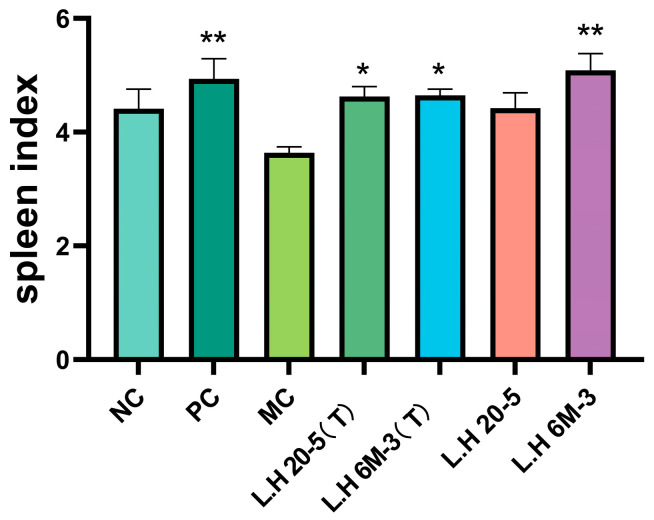
Effects of CTX and extracellular polysaccharide interventions on murine immunity. Note: NC: negative control group; PC: positive control group; MC: model group; L.H 20-5(T): fucose-type L.H 20-5 group; L.H 6M-3(T): mannose-type L.H 6M-3 group; L.H 20-5: rhamnose-type L.H 20-5 group; L.H 6M-3: xylose-type L.H 6M-3 group. Compared with the model group, * *p* < 0.05, ** *p* < 0.01.

The results of the animal spleen index are shown in Figure 1. Compared to the MC group, the extracellular polysaccharide (EPS) intervention groups demonstrated varying degrees of spleen index enhancement. Compared with the MC group, the exopolysaccharide group (L.H 20-5) produced by *Lactobacillus helveticus* DYNDL_20-5 cultured without fucose could not significantly increase the spleen index (*p* > 0.05). In contrast, the fucose-type exopolysaccharide group (L.H 20-5(T)) produced by *Lactobacillus helveticus* DYNDL_20-5 cultured with the addition of fucose could significantly increase the spleen index (*p* < 0.05). Meanwhile, the mannose-type exopolysaccharide group (L.H 6M-3(T)) produced by *Lactobacillus helveticus* 6M-3 cultured with the addition of mannose was also able to significantly increase the spleen index (*p* < 0.05). These results indicate that the exopolysaccharides produced by lactobacilli cultured with the exogenous addition of mannose or fucose improved the spleen index.

### 3.3. Effects of Different Extracellular Polysaccharide Interventions on Murine Colon

**Figure 2:**
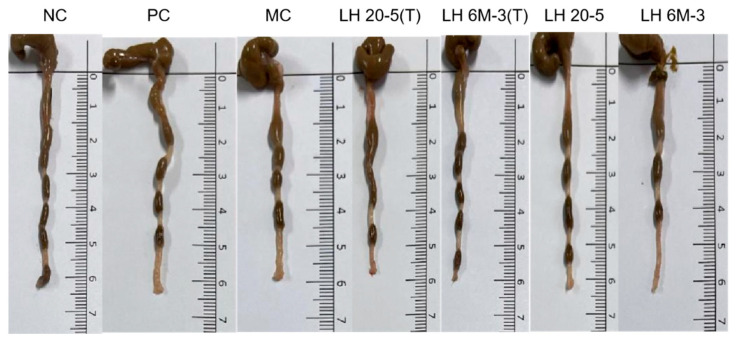
Effects of different extracellular polysaccharide interventions on murine colon Note: NC: negative control group; PC: positive control group; MC: model group; L.H 20-5(T): fucose-type L.H 20-5 group; L.H 6M-3(T): mannose-type L.H 6M-3 group; L.H 20-5: rhamnose-type L.H 20-5 group; L.H 6M-3: xylose-type L.H 6M-3 group.

Colon length serves as a preliminary macroscopic indicator for evaluating intestinal barrier function. Measurements of colon length across experimental groups (Figure 2) revealed that compared to the NC group, the MC group exhibited a slight tendency towards reduced colon length, although no significant difference was observed. CTX-induced immunodeficiency may exert a mild potential impact on the intestinal barrier, but did not cause significant structural damage to the colon. Further comparison of colon length between EPS intervention groups and the MC group revealed that neither conventionally cultured EPS (L.H 20-5 group, L.H 6M-3 group) nor monosaccharide-optimised EPS (L.H 20-5(T) group, L.H 6M-3(T) group) produced significant differences in colon length.

### 3.4. Effects of Different Types of Exopolysaccharide Interventions on Mouse Cytokines

This study employed enzyme-linked immunosorbent assay (ELISA) to quantify cytokine levels in serum (systemic immunity) and small intestinal tissue (local mucosal immunity), investigating the remodelling effects of exopolysaccharides on the cytokine network in immunodeficient mice.

#### 3.4.1. Effects of Different Types of Exopolysaccharide Interventions on Serum Cytokines

Serum cytokines reflect the overall immune activation status of the organism [16,17].

**Figure 3:**
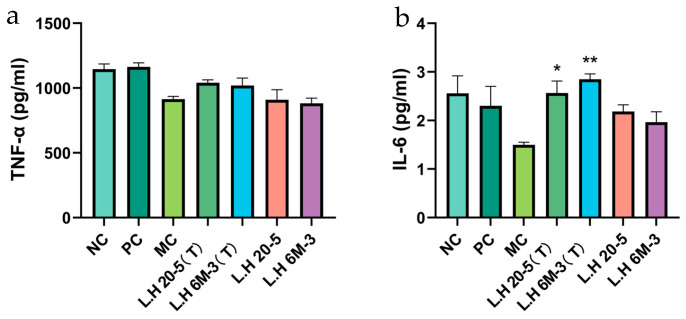

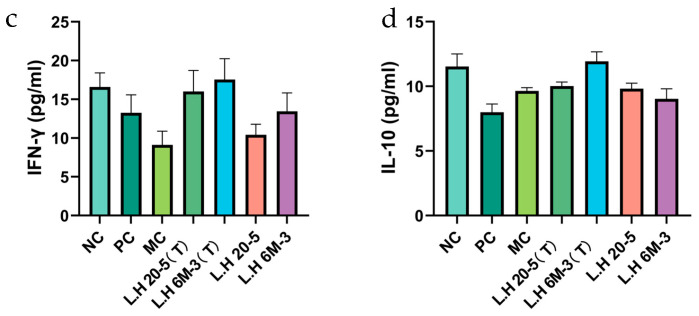
Effects of different exopolysaccharide interventions on serum cytokines. Note: (**a**) TNF-α; (**b**) IL-6; (**c**) IFN-γ; (**d**) IL-10. NC: negative control group; PC: positive control group; MC: model group; L.H 20-5(T): fucose-type L.H 20-5 group; L.H 6M-3(T): mannose-type L.H 6M-3 group; L.H 20-5: rhamnose-type L.H 20-5 group; L.H 6M-3: xylose-type L.H 6M-3 group. Compared with the model group, * *p* < 0.05, ** *p* < 0.01.

Compared with the model group (MC group), the exopolysaccharide group produced by *Lactobacillus helveticus* DYNDL_20-5 cultured without fucose (L.H 20-5) and the exopolysaccharide group produced by *Lactobacillus helveticus* 6M-3 cultured without mannose (L.H 6M-3) did not cause significant changes in the level of tumour necrosis factor-α (TNF-α) (*p* > 0.05). In contrast, the fucose-type exopolysaccharide group produced by *Lactobacillus helveticus* DYNDL_20-5 cultured with the addition of fucose (L.H 20-5(T)) and the mannose-type exopolysaccharide group produced by *Lactobacillus helveticus* 6M-3 cultured with the addition of mannose (L.H 6M-3(T)) slightly increased the TNF-α level, but no significant difference was observed (*p* > 0.05) (Figure 3a). Compared with the model group (MC group), the levels of interleukin-6 (IL-6) in the L.H 20-5 group and L.H 6M-3 group showed no significant changes (*p* > 0.05); however, the IL-6 levels in the fucose-type L.H 20-5 group (L.H 20-5(T)) was significantly elevated (*p* < 0.05), mannose-type L.H 6M-3 group (L.H 6M-3(T)) was significantly elevated (*p* < 0.01) (Figure 3b). In comparison to the model group (MC group), the levels of interferon-γ (IFN-γ) in the L.H 20-5 group and L.H 6M-3 group were slightly increased, but no significant difference was observed (*p* > 0.05). Moreover, the IFN-γ levels in the fucose-type L.H 20-5 group (L.H 20-5(T)) and mannose-type L.H 6M-3 group (L.H 6M-3(T)) were higher than those in the L.H 20-5 group and L.H 6M-3 group (Figure 3c). Compared with the model group (MC group), the level of interleukin-10 (IL-10) in the L.H 6M-3 group did not change significantly (*p* > 0.05), while the IL-10 level in the mannose-type L.H 6M-3 group (L.H 6M-3(T)) was increased, but no significant difference was observed (*p* > 0.05) (Figure 3d). These results indicate that the fucose-type exopolysaccharides from L.H 20-5 and mannose-type exopolysaccharides from L.H 6M-3 can promote the secretion levels of cytokines in immune-damaged mice, thereby enhancing the immune function of the mice.

#### 3.4.2. Effects of Different Types of Exopolysaccharide Interventions on Cytokines in the Small Intestine

**Figure 4:**
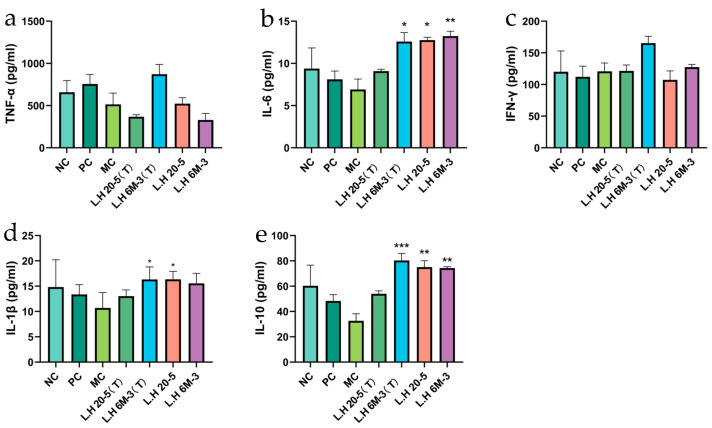
Effects of different exopolysaccharide interventions on cytokines in the small intestine. Note: (**a**) TNF-α; (**b**) IL-6; (**c**) IFN-γ; (**d**) IL-1β; (**e**) IL-10. NC: negative control group; PC: positive control group; MC: model group; L.H 20-5(T): fucose-type L.H 20-5 group; L.H 6M-3(T): mannose-type L.H 6M-3 group; L.H 20-5: rhamnose-type L.H 20-5 group; L.H 6M-3: xylose-type L.H 6M-3 group. Compared with the model group, * *p* < 0.05, ** *p* < 0.01, *** *p* < 0.001.

As the primary site of mucosal immunity, the small intestine reflects local intestinal immune function through its cytokine profile. We further examined cytokine expression in the small intestine. ELISA analysis revealed that, compared with the model group (MC group), the mannose-type L.H 6M-3 group (L.H 6M-3(T)) exhibited a stronger effect in increasing the TNF-α level than the L.H 6M-3 group (Figure 4a). Additionally, the IL-6 level in the mannose-type L.H 6M-3 group (L.H 6M-3(T)) was significantly higher than that in the model group (Figure 4b). Compared with the model group (MC group), the IFN-γ level in the L.H 6M-3 group showed no significant change (*p* > 0.05). The IFN-γ level in the mannose-type L.H 6M-3 group (L.H 6M-3(T)) was increased, but no significant difference was observed (*p* > 0.05) (Figure 4c). In comparison to the model group (MC group), the levels of IL-1β in the mannose-type L.H 6M-3 group (L.H 6M-3(T)) were significantly elevated (*p* < 0.05) (Figure 4d), and the levels of IL-10 in the mannose-type L.H 6M-3 group (L.H 6M-3(T)) were significantly elevated (*p* < 0.001) (Figure 4e). The above results indicate that the mannose-type exopolysaccharides from L.H 6M-3 can promote the secretion levels of cytokines in immune-damaged mice, thereby enhancing the immune function of the mice.

### 3.5. Effects of Different Types of Exopolysaccharide Interventions on Spleen Pathology

**Figure 5:**
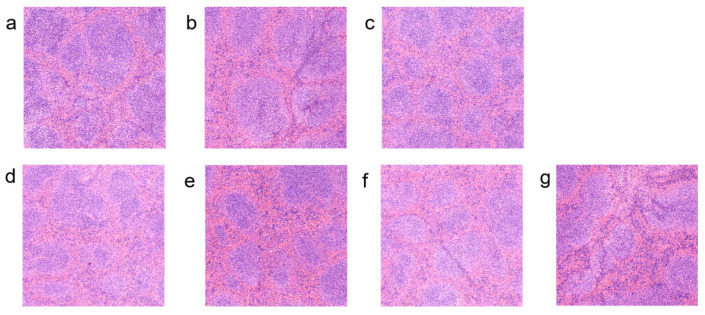
Effects of different exopolysaccharide interventions on spleen pathology. Legend: (**a**) NC; (**b**) PC; (**c**) MC; (**d**) L.H 20-5(T); (**e**) L.H 6M-3(T); (**f**) L.H 20-5; (**g**) L.H 6M-3 (Scale bar = 100 μm). NC: negative control group; PC: positive control group; MC: model group; L.H 20-5(T): fucose-type L.H 20-5 group; L.H 6M-3(T): mannose-type L.H 6M-3 group; L.H 20-5: rhamnose-type L.H 20-5 group; L.H 6M-3: xylose-type L.H 6M-3 group.

This study examined pathological changes in the spleens of mice across different groups via haematoxylin and eosin (HE) staining to investigate the role of EPS in repairing the structure of immune organs. Experimental results showed that in the NC group (Figure 5a), the spleen structure was intact, the white pulp region was clearly defined, and the boundary between red and white pulp was distinct, indicating normal immune function. In the PC group (Figure 5b), the white pulp area expanded compared with the MC group, whilst the red pulp area contracted, indicating that thymosin can repair spleen structure. In the MC group (Figure 5c), the spleen’s white pulp area showed significant atrophy with relative enlargement of the red pulp area, suggesting that cyclophosphamide (CTX) leads to suppressed T/B cell proliferation and diminished immune response capacity. Compared with the MC group, the intervention groups treated with fucose-type L.H 20-5 (L.H 20-5(T)) (Figure 5d) and L.H 6M-3 (Figure 5g) exhibited enlarged white pulp areas and reduced red pulp areas in the spleen, indicating that these two exopolysaccharides significantly promoted immune responses. Mannose-type L.H 6M-3 (L.H 6M-3(T)) (Figure 5e) and L.H 20-5 (Figure 5f) did not cause significant morphological changes in the spleen.

### 3.6. Effects of Different Types of Exopolysaccharide Interventions on Colon Pathology

**Figure 6:**
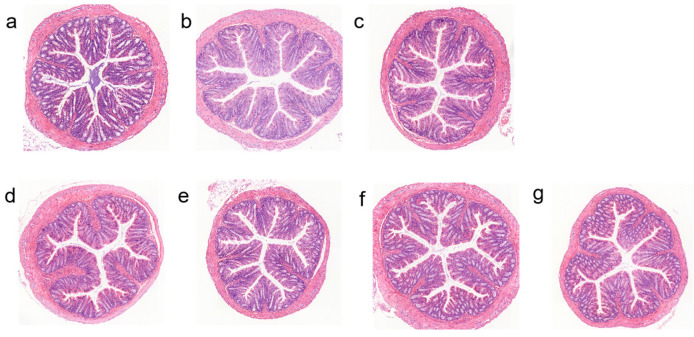
Effects of different exopolysaccharide interventions on colon pathology. Legend: (**a**) NC; (**b**) PC; (**c**) MC; (**d**) L.H 20-5(T); (**e**) L.H 6M-3(T); (**f**) L.H 20-5; (**g**) L.H 6M-3 (Scale bar = 100 μm). NC: negative control group; PC: positive control group; MC: model group; L.H 20-5(T): fucose-type L.H 20-5 group; L.H 6M-3(T): mannose-type L.H 6M-3 group; L.H 20-5: rhamnose-type L.H 20-5 group; L.H 6M-3: xylose-type L.H 6M-3 group.

Histopathological changes in the colons of mice across groups were examined via HE staining to evaluate the protective effect of EPS on intestinal barrier structure. Experimental results showed (Figure 6) that, compared with the MC group, all exopolysaccharide intervention groups—fucose-type L.H 20-5 (L.H 20-5(T)), mannose-type L.H 6M-3 (L.H 6M-3(T)), L.H 20-5, and L.H 6M-3—exhibited no significant differences in colonic crypt depth, goblet cell count, or inflammatory cell infiltration.

### 3.7. Effects of Different Types of Exopolysaccharides on Liver Pathology

**Figure 7:**
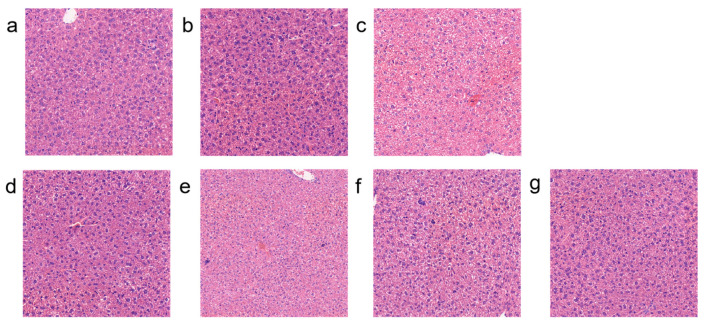
Effects of different exopolysaccharide interventions on liver pathology. Legend: (**a**) NC; (**b**) PC; (**c**) MC; (**d**) L.H 20-5(T); (**e**) L.H 6M-3(T); (**f**) L.H 20-5; (**g**) L.H 6M-3 (Scale bar = 20 μm). NC: negative control group; PC: positive control group; MC: model group; L.H 20-5(T): fucose-type L.H 20-5 group; L.H 6M-3(T): mannose-type L.H 6M-3 group; L.H 20-5: rhamnose-type L.H 20-5 group; L.H 6M-3: xylose-type L.H 6M-3 group.

Pathological changes in the livers of mice in each group were observed through HE staining to investigate the protective effect of EPS on liver damage in immunodeficient mice. As shown in Figure 7, the model group mice exhibited more severe liver damage, primarily manifested as hepatic vacuolar degeneration. In contrast, fucose-type L.H 20-5 (L.H 20-5(T)), mannose-type L.H 6M-3 (L.H 6M-3(T)), and L.H 6M-3 reduced hepatic lipid vacuolisation. This indicates that exogenously supplemented fucose-type and mannose-type exopolysaccharides can mitigate hepatocellular damage in mice.

### 3.8. Effects of Different Types of Exopolysaccharide Interventions on Gut Microbiota

**Figure 8:**
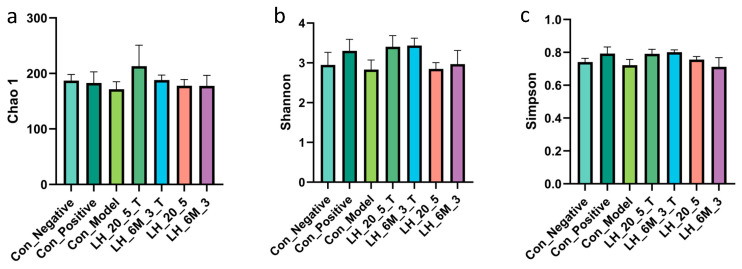

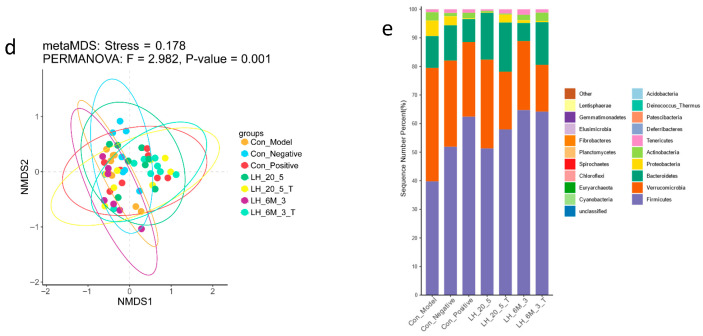
Effects of different types of exopolysaccharide interventions on gut microbiota: (**a**) Chao1 index; (**b**) Shannon index; (**c**) Simpson index; (**d**) beta diversity analysis; (**e**) gut microbiota composition. Con_Negative: Negative control group; Con_Positive: Positive control group; Con_Model: model group; L.H_20_5_T: fucose-type L.H 20-5 group; L.H_6M_3_T: mannose-type L.H 6M-3 group; L.H_20_5: rhamnose-type L.H 20-5 group; L.H_6M_3: xylose-type L.H 6M-3 group.

The α-diversity of gut microbiota can be assessed using the Chao1 index, which reflects species richness, and the Shannon and Simpson indices, which reflect species diversity [18,19]. As shown in Figure 8a–c, compared with the Con_Negative group, the Con_Model group exhibited reduced Chao1, Shannon, and Simpson indices, indicating decreased gut microbiota diversity. Relative to the Con_Model group, there were no significant changes in the Chao 1, Shannon, and Simpson indices in the L.H 20-5 group and L.H 6M-3 group (*p* > 0.05). However, the Chao 1, Shannon, and Simpson indices all increased in the fucose-type L.H 20-5 group (L.H 20-5(T)) and the mannose-type L.H 6M-3 group (L.H 6M-3(T)). These results indicate that the exopolysaccharides of fucose-type L.H 20-5 group (L.H 20-5(T)) and mannose-type L.H 6M-3 group (L.H 6M-3(T)) enhance the α-diversity of the intestinal flora in mice. As shown in Figure 8d, the results of beta diversity analysis indicated that there were significant differences in the gut microbiota among different groups.

At the phylum level, the microbial community structures of faecal samples from each group comprised Firmicutes, Verrucomicrobia, Bacteroidetes, Proteobacteria, Actinobacteria, and Tenericutes. Amongst these, Firmicutes, Verrucomicrobia, and Bacteroidetes were the dominant microbial groups. Comparing the relative abundances of bacterial phyla across groups, the Con_Model group showed no significant changes in the abundances of Firmicutes, Verrucomicrobia, and Bacteroidetes compared with the Con_Negative and Con_Positive groups. Compared with the Con_Model group, the abundance of Firmicutes significantly increased following intervention with fucose-type L.H 20-5 (L.H 20-5(T)) and mannose-type L.H 6M-3 (L.H 6M-3(T)). As shown in Figure 8e, at the phylum level, both mannose-type and fucose-type EPS significantly influence the composition of the murine gut microbiota.

**Figure 9:**
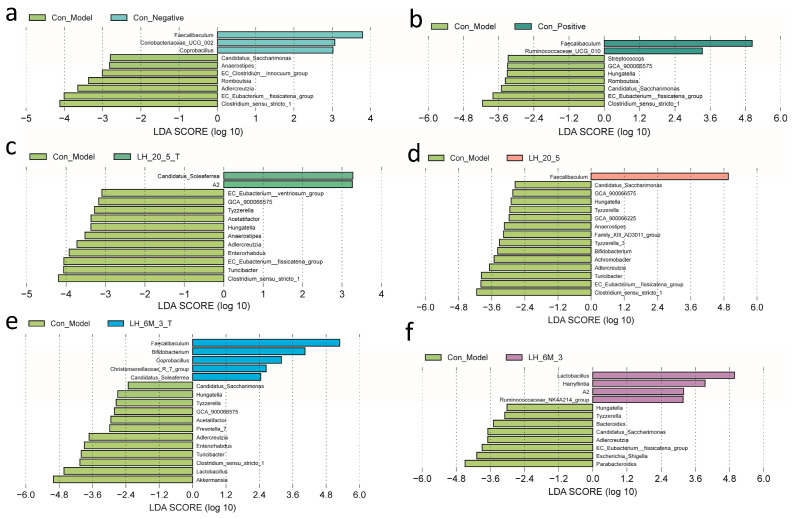
Analysis of gut microbiota at the genus level.(**a**) Con_Model vs Con_Negative; (**b**) Con_Model vs Con_Positive; (**c**) Con_Model vs L.H_20_5_T; (**d**) Con_Model vs L.H_20_5; (**e**) Con_Model vs L.H_6M_3_T; (**f**) Con_Model vs L.H_6M_3.Note: Con_Negative: negative control group; Con_Positive: positive control group; Con_Model: model group; LH_20_5_T: fucose-type L.H 20-5 group; LH_6M_3_T: mannose-type L.H 6M-3 group; LH_20_5: rhamnose-type L.H 20-5 group; LH_6M_3: xylose-type L.H 6M-3 group.

To further compare the differences in gut microbiota among various groups, LEfSe analysis was performed. As shown in Figure 9a–f, the differential species between the model group and each of the other groups were compared. The model group was mainly enriched in Clostridium, a genus that includes pathogenic species capable of exerting adverse effects. The negative control group and positive control group were predominantly enriched in Faecalibaculum, a genus with anti-inflammatory properties that protects the digestive system from intestinal pathogens. The mannose-type L.H 6M-3 (L.H 6M-3(T)) group was mainly enriched in Bifidobacterium and Faecalibaculum. Bifidobacterium can maintain gut microbiota balance and enhance immune system function. These results indicate that the exopolysaccharides of mannose-type L.H 6M-3 (L.H 6M-3(T)) group can increase the levels of beneficial bacterial genera.

## 4. Discussion

Immunodeficiency, a common complication arising from clinical treatments such as tumour radiotherapy, chemotherapy, and organ transplantation, severely impairs patient rehabilitation and quality of life [20,21]. Cyclophosphamide (CTX), a typical immunosuppressant, whilst effectively inhibiting abnormal immune responses, induces a series of adverse effects including immune organ atrophy, cytokine network imbalance, and intestinal flora disturbance [22,23,24]. Current clinical interventions predominantly rely on chemical agents, which are characterised by single-target mechanisms and significant side effects. Consequently, the development of natural immunomodulators with multi-target activity and high safety profiles has become increasingly urgent. This study focused on exopolysaccharides (EPS) from *Lactobacillus helveticus* containing mannose or fucose, systematically evaluating their immunomodulatory effects in a CTX-induced immunodeficient mouse model and exploring their mechanisms of action across multiple dimensions, including immune organs, cytokines, histopathology, and intestinal microbiota.

The spleen, as the largest peripheral immune organ, serves as a critical site for the congregation, activation, and immune response mediation of T lymphocytes, B lymphocytes, and macrophages. The spleen index (spleen-to-body weight ratio) represents a key quantitative indicator reflecting immune organ development and functional status [25,26]. Splenic pathological morphology directly reflects immune function: the white pulp, comprising periarteriolar lymphoid sheaths and lymphoid follicles, constitutes the lymphocyte aggregation zone responsible for initiating immune responses, whilst the red pulp, containing splenic cords and sinuses, participates in blood cell filtration and immune cell activation [27]. As a classical alkylating agent immunosuppressant, CTX inhibits lymphocyte DNA synthesis and proliferation, leading to immune organ atrophy and functional impairment, manifested by significant spleen index reduction. This study demonstrated that EPS cultured with specific monosaccharide optimisation significantly increased the spleen index in immunodeficient mice and ameliorated white pulp atrophy. As the core peripheral immune organ, splenic structural integrity directly determines T and B lymphocyte activation capacity and their ability to mediate immune responses. White pulp expansion suggests that EPS may promote lymphocyte homing and proliferation, thereby enhancing both cellular and humoral immunity. Notably, fucose- or mannose-optimised EPS exhibited superior performance in improving splenic structure and function, which is closely associated with sugar chain conformational changes potentially induced by monosaccharide composition modification. Previous studies have indicated that polysaccharide immunomodulatory activity is highly dependent on glycosidic bond types, branching structures, and monosaccharide compositions [28,29,30]. The present findings further support this perspective and provide experimental evidence for directional enhancement of EPS immunological activity through medium optimisation strategies.

Cytokines, functioning as intercellular signalling molecules, directly determine immune response intensity and direction through their expression levels and balance [31,32]. Tumour necrosis factor-α (TNF-α), interleukin-6 (IL-6), and interferon-γ (IFN-γ) are core pro-inflammatory factors participating in pathogen clearance and inflammation initiation; however, their overexpression can precipitate immune imbalance. Conversely, interleukin-10 (IL-10), a major anti-inflammatory factor, inhibits excessive inflammatory responses and maintains immune homeostasis [33]. This study employed enzyme-linked immunosorbent assay (ELISA) to quantify these cytokines in serum (systemic immunity) and small intestinal tissue (local mucosal immunity), thereby exploring EPS-mediated cytokine network remodelling in immunodeficient mice. The results revealed that EPS could restore cytokine balance in both systemic and mucosal immunity. This suggests that whilst promoting inflammatory response initiation, EPS simultaneously prevents excessive inflammatory damage through anti-inflammatory factor upregulation, reflecting its bidirectional immunomodulatory properties. This balanced restoration is particularly crucial for immunocompromised organisms, as it ensures effective pathogen clearance whilst preventing secondary tissue damage from cytokine storms. We found that fucose-optimised EPS restored the cytokine balance in serum but had no significant effect locally in the small intestinal tissue. This discrepancy could be due to the following: 1. Differences in timing or sensitivity between local and systemic immune responses: The mucosal immune microenvironment of the small intestine involves a more complex cytokine network, regulated by local factors, diverse cell populations, and distinct signalling pathways. 2. We hypothesize that the polysaccharides may primarily target other organs, a possibility that requires further investigation.

The liver, as a vital organ for metabolism and immune regulation, is susceptible to cytotoxic damage from CTX metabolites (such as acrolein). A common pathological manifestation is hepatocellular fatty vacuolar degeneration (lipid metabolism disorder), which can compromise hepatic immune function in severe cases. Haematoxylin-eosin (H&E) staining was employed to observe hepatic pathological changes across experimental groups, thereby exploring EPS’s protective effects against liver damage in immunodeficient mice. At the histopathological level, EPS-mediated amelioration of hepatocellular fatty vacuolar degeneration warrants particular attention. The liver functions not only as a metabolic centre but also as an important site for immune cell residence and activation [34,35,36]. CTX metabolite-induced hepatocellular damage may further exacerbate systemic immunosuppression, whilst EPS indirectly supports hepatic immunomodulatory function by reducing hepatocellular steatosis.

The intestinal flora, known as the “second genome”, exerts profound effects on immune homeostasis through its structural and functional stability [37,38]. Through sequencing analysis, this study demonstrated that CTX-induced immunodeficiency decreased intestinal flora diversity and disrupted microbial community structure in mice. However, EPS intervention increased α-diversity, enhanced the relative abundance of Firmicutes, and promoted the proliferation of beneficial genera, including Faecalibacterium, and Bifidobacterium. These microbial alterations are closely associated with immune function improvement: Firmicutes contribute to short-chain fatty acid production, which plays a pivotal role in regulating regulatory T (Treg) cell differentiation and suppressing inflammatory responses; Bifidobacterium and Faecalibacterium are widely recognised probiotic genera with anti-inflammatory and immunoenhancing properties. These findings indicate that EPS can partially reverse CTX-induced dysbiosis and restore normal flora-immune axis function. It should be noted that faecal samples were used to evaluate the gut microbiota in this study. Although the faecal microbiome is a convenient and commonly used indicator for assessing gut microbial profiles, other research findings have shown that it may not fully represent the entire gut microbiome [39]. Based on the analysis results of faecal samples, whether EPS exerts immunomodulatory effects by regulating the microbiota in specific segments of the gut (such as the surface of the small intestinal mucosa) cannot be directly verified with the current data, which warrants further investigation.

In conclusion, this study confirmed through multiple perspectives the comprehensive benefits of mannose-type and fucose-type *Lactobacillus helveticus* EPS in ameliorating immunodeficiency. The underlying mechanisms involve coordinated effects across multiple pathways, including immune organ structural repair, cytokine network remodelling, intestinal flora homeostasis regulation, and hepatoprotection. Compared with EPS cultured under standard conditions, monosaccharide-optimised EPS exhibited superior biological activity across multiple indicators, highlighting the critical role of monosaccharide composition in EPS functional optimisation.

This study has certain limitations. The regulatory mechanisms of EPS on key immune signalling pathways such as MAPK and JAK/STAT were not thoroughly explored, nor was a clear dose–response relationship established. Future studies could integrate transcriptomics and proteomics technologies to further elucidate the molecular targets of EPS; simultaneously, through optimisation of different doses and administration regimens, the optimal intervention strategy could be clarified, providing more robust theoretical foundations for developing Lactobacillus EPS in functional foods and clinical adjuvant therapy.

## 5. Conclusions

This study employed a CTX-induced immunodeficient mouse model to systematically investigate the immunomodulatory effects of extracellular polysaccharides from different *Lactobacillus helveticus* strains. Results indicate that both mannose-type and fucose-type extracellular polysaccharides significantly increased the spleen index in model mice, modulated levels of pro-inflammatory cytokines, including TNF-α, IL-6, and IFN-γ in serum and small intestine, and elevated IL-10 expression levels. They also ameliorated splenic white pulp atrophy and hepatic vacuolar degeneration. Concurrently, they modulated gut microbiota composition by increasing diversity and elevating the abundance of phyla such as Firmicutes, as well as genera including Bacteroides, Faecalibacterium, and Bifidobacterium. In summary, EPS produced by *Lactobacillus helveticus* cultivated in media supplemented with mannose or fucose enhanced immune function in immunodeficient mice through multiple pathways by regulating immune organ pathology, cytokine levels, and gut microbiota, thereby providing a theoretical foundation for the development and application of Lactobacillus extracellular polysaccharides in immunomodulating functional foods.

## Figures and Tables

**Table 1:** Effect of fucose supplementation on the monosaccharide composition of *Lactobacillus helveticus* extracellular polysaccharides (molar percentage).

*Lactobacillus* Strain	Carbon Source	Glucose	Galactose	Rhamnose	Fucose	Mannose	Xylose	Arabinose
*L. helveticus* DYNDL 20-5	Glucose	46.23	14.06	5.72	0	0	0	11.48
*L. helveticus* DYNDL 20-5	Fucose	35.03	8.90	0	10.46	0	10.97	15.99

**Table 2:** Effect of mannose supplementation on the monosaccharide composition of *Lactobacillus helveticus* extracellular polysaccharides (molar percentage).

*Lactobacillus* Strain	Carbon Source	Glucose	Galactose	Rhamnose	Fucose	Mannose	Xylose	Arabinose	Glucosamine	Galactosamine	Galacturonic Acid
*L. helveticus* 6M-3	Glucose	17.63	9.44	0	0	0	57.1	0	4.75	7.89	3.22
*L. helveticus* 6M-3	Mannose	13	11.22	0	0	5.9	53.56	0	6.61	9.71	0

## Data Availability

The original contributions presented in this study are included in the article. Further inquiries can be directed to the corresponding authors.

## Abbreviations

EPS: exopolysaccharides; CTX: cyclophosphamide; NC: negative control group; PC: positive control group; MC: model group; TNF-α: tumour necrosis factor-alpha; IL-6: interleukin-6; IFN-γ: interferon-gamma; IL-10: interleukin-10; HE: haematoxylin and eosin; OTUs: operational taxonomic units; PCoA: principal coordinate analysis.
